# Data-independent acquisition quantitative proteomics analysis of milk fat globule membrane proteins in rabbit colostrum and mature milk

**DOI:** 10.3389/fvets.2026.1703387

**Published:** 2026-03-19

**Authors:** Liangde Kuang, Min Lei, Xueer Mu, Yunduan Wang, Yueyue Li, Hongmei Xu, Tiantian Li, Lin Huang, Yucai Zheng, Xiaohong Xie, Congyan Li, Wei Fu

**Affiliations:** 1 Animal Genetic Breeding and Reproduction Key Laboratory of Sichuan Province, Sichuan Animal Science Academy, Chengdu, China; 2 Key Laboratory of Qinghai-Tibetan Plateau Animal Genetic Resource Reservation and Utilization, Ministry of Education, Southwest Minzu University, Chengdu, China; 3 Academy of National Food and Strategic Reserves Administration, Beijing, China

**Keywords:** colostrum, DIA, milk fat globule membrane, proteomics, rabbit

## Abstract

Proteomics has been widely used to identify proteins in the milk fat globule membrane (MFGM). However, the characteristics of MFGM proteins in rabbit colostrum (RC) and mature milk (RM) remain largely unexplored. This study aimed to profile the rabbit MFGM proteins and assess differences in component and functional profiles between RC and RM through data-independent acquisition (DIA) quantitative proteomics. We established the proteomic profile of rabbit MFGM, identifying 3,548 proteins across RC and RM. Notably, typical MFGM proteins, such as Perilipin 2 (PLIN2), Xanthine dehydrogenase/oxidase (XDH/XO), and Apolipoprotein, were detected in rabbit milk, and 10 of these were confirmed by Parallel reaction monitoring (PRM) detection. Comparative analysis revealed 480 differentially expressed MFGM proteins (DEMPs), with 379 up-regulated and 101 down-regulated DEMPs in RC compared to RM. This included 68 unique proteins in RC, 5 in RM, and 407 DEMPs expressed in both groups. The GO analysis indicated that DEMPs are predominantly involved in processes such as proteolysis, cell adhesion, and ion transport, with enrichments of 32, 14, and 14 DEMPs, respectively. KEGG analysis gathered 56 significant pathways, most of which were categorized into Human Diseases (20/56) and Metabolism (14/56). Protein–protein interaction (PPI) network analysis emphasized the core role of DEMPs (e.g., proteasome subunits and integrins) in human diseases (e.g., Alzheimer’s disease) and in signal transduction (e.g., the PI3K-Akt signaling pathway). These results offer theoretical insights into the components and functions of rabbit milk, suggesting a novel way to enhance the economic benefit of the rabbit industry.

## 1 Introduction

Milk is a complex fluid composed of a wide array of essential nutrients required by neonatal mammals, including proteins, amino acids, lipids, carbohydrates, vitamins, and minerals. The nutrients in milk, especially those found in colostrum, play a vital and irreplaceable role in the early establishment of the immune system, colonization of the gut microbiota, and the development of the nervous system and cognitive functions in newborns (1, 2). Colostrum is characterized by exceptionally high concentrations of bioactive components and essential nutrients, which are crucial for meeting the immediate nutritional demands of neonates and for mediating the transfer of passive immunity during the early postnatal period (3). As lactation progresses, the levels of immunologically active substances and other bioactive compounds decline markedly in transitional milk, ultimately reaching concentrations comparable to those observed in late lactation milk (4, 5). Importantly, the composition of milk is not static; instead, it exhibits dynamic variation influenced by multiple factors, including breed, lactation stage, species differences, and maternal nutritional status (6). For mothers who are unable to breastfeed due to some constraints, infant formula provides a vital nutritional alternative to ensure proper growth and health maintenance in early life (7).

Milk proteins and fats are the main nutrients in milk. Casein and whey protein are the predominant lactoproteins in milk, but a variety of other milk proteins also exist, many of which have significant functional roles (8). The lipids in milk, primarily composed of triglycerides, exhibit hydrophobic properties and form milk fat globules (MFGs). These globules are secreted by mammary epithelial cells and are wrapped in a three-layered phospholipid structure, known as the milk fat globule membrane (MFGM) (9). In recent decades, studies have identified a multitude of proteins that are either inserted or adhere to the MFGM, collectively referred to as MFGM proteins. These proteins have been confirmed to play essential roles in a range of biological processes, including cell growth and differentiation, immune defense, and lipid metabolism (10, 11). Although MFGM proteins merely account for 1% ~ 4% of the whole milk proteins, they have already been observed by immunofluorescence staining technology and isolated from various mammals’ milk (human, cattle, pig, goat, donkey, mare, camel, yak, etc.) (10–12), implying their irreplaceable role in an infant’s development.

Cow milk is the predominant source of dairy products for humans, accounting for approximately 80% of global milk production annually (13). However, with the continued advancement of analytical technologies, the identification of low-abundance bioactive components has also underscored the inherent limitations of cow milk. As a result, alternative milk sources, such as yak, buffalo, mare, donkey, goat, and camel milk, have gained increasing attention in specific regions for their distinctive nutritional properties and market potential (14, 15). Most importantly, these scarce substitutes provide the special nutrients that supplement the choices of dairy products for special customers (e.g., lactose intolerance, milk allergy), thereby enriching the diversity of dairy options available to the public (16). The fatty acid profile of cow milk is primarily dominated by saturated fatty acids (SFA), with comparatively low levels of monounsaturated (MUFA) and polyunsaturated fatty acids (PUFA), comprising 55.0–73.0% SFA, 2.0–30.0% MUFA, and 2.4–6.3% PUFA (17). Conversely, rabbit milk contains 70.4% SFA, 12.8% MUFA, and 9% PUFA, indicating a significantly higher proportion of MUFA and PUFA compared to cow milk (18). The enrichment of MUFAs and PUFAs in rabbit milk suggests an increased demand for coordinated lipid trafficking, membrane remodeling, and antioxidant protection during milk fat secretion. Moreover, the average diameter of MFGs in rabbit milk is approximately 5.8 μm, which is larger than that in cow milk (3.2–4.5 μm) (18, 19). The size of MFGs plays a critical role in determining the stability, functionality, and nutritional value of milk, as larger MFGs are more prone to coagulation during processing. Consequently, variations in MFG diameter and lipid unsaturation are expected to be closely associated with alterations in the composition and functional organization of MFGM proteins. However, studies on rabbit milk remain scarce, with only a limited number of reports addressing the chemical composition and morphological characteristics of rabbit colostrum (RC) and mature milk (RM) (20). Therefore, elucidating and comparing the proteomic profiles of RC and RM is essential for improving our understanding of protein composition and biological function across different lactation stages. Incorporating the bioactive components of rabbit milk into infant formula alternatives may offer considerable benefits for functional nutrition.

Advances in proteomic techniques, such as tandem mass tag (TMT) (21), isobaric tags for relative and absolute quantitation (iTRAQ) (22), data-independent acquisition (DIA) proteomics (23), and label-free quantitative proteomics (LFQP) (10), have been broadly used to determine the composition of MFGM proteins in mammal milk. These techniques have been extensively applied to study the constituents of MFGM proteins and their post-translational modifications (PTMs) between colostrum and RM in different species such as human, cattle, goat, donkey, and swine (11, 24–26). For example, using mass spectroscopy-based *N*-glycoproteomics, Cao et al. (24) identified 912 *N*-glycosylation sites on 506 *N*-glycoproteins in human colostrum and RM MFGM, with 220 *N*-glycoproteins containing 304 *N*-glycosylation sites differentially expressed in them. Functional enrichment analysis revealed that the majority of differentially expressed N-glycoproteins were closely associated with phagosome-related processes, cell adhesion molecule pathways, and several disease-related signaling pathways. Another study identified 3,917 and 3,966 MFGM proteins in porcine colostrum and mature milk, respectively, including 303 significantly differentially expressed MFGM proteins (DEMPs) (25). Functional annotation indicated that the DEMPs were predominantly associated with cellular processes, cellular components, and molecular functions related to binding activities. KEGG pathway enrichment further revealed that these proteins were mainly involved in phagosome-associated pathways. These studies illustrate that while animal milk serves as a valuable substitute for human milk, there are significant variations in the composition of MFGM proteins among different species (10). Most importantly, the results imply that with a comprehensive understanding of the protein composition and functions in animal milk, we can potentially customize dairy products to human preferences using gene-edited mammary gland bioreactors (27). However, the specific composition of rabbit milk MFGM proteins and the differences between colostrum and RM remain unclear. Nonetheless, a comprehensive characterization of rabbit MFGM proteins provides a mechanistic foundation for the rational design of rabbit milk substitutes. Given the distinctive features of rabbit milk, including elevated PUFA content and larger milk fat globules, elucidating the corresponding MFGM protein composition is essential for understanding how lipid packaging, membrane stability, and nutrient delivery are coordinated during lactation. Such knowledge can inform the optimization of fat structure and bioactive protein components in milk replacers, thereby improving lipid digestibility, immune support, and early postnatal adaptation in newborn rabbits. From an applied perspective, these improvements are directly relevant to reducing neonatal mortality associated with artificial feeding and enhancing nutritional strategies for pet rabbits (28), ultimately supporting the sustainability and performance of the rabbit industry. Additionally, by comparing the milk protein components across species, we can enhance our understanding of milk composition in various animals. This knowledge provides a theoretical foundation for the use of mammary gland bioreactors to produce high-quality milk proteins that meet human nutritional needs.

In the present study, we collected RC and RM (days 1 and 17 postpartum). We then isolated the MFGM proteins and assessed their separation by SDS-PAGE. This approach allowed us, for the first time, to delineate the MFGM protein profile in rabbit milk and to conduct a comparative analysis of the proteins between RC and RM using DIA quantitative proteomics. For the DEMPs, we conducted the GO and KEGG analyses to acquire the core DEMPs and their corresponding pathways. Finally, using PRM technology, we further confirmed the reliability and repeatability of the proteomic data in this study. Our research presents the first comprehensive protein profile of the MFGM in rabbit milk. It offers a detailed comparative analysis between RC and RM, providing the theoretical basis for understanding the components and function of rabbit milk.

## 2 Materials and methods

The experiment was conducted according to the National Institutes of Health (NIH) Guidelines and the National Research Council’s publication “Guide for Care and Use of Laboratory Animals” and approved by the Animal Care and Use Committee of the Sichuan Animal Science Academy (Approval code: 2023020).

### 2.1 Sample collection of rabbit colostrum and mature milk

Thirty-six rabbit milk samples were collected from 18 parous meat rabbits of Shu Xing No.1 (1.5 years old, body weight: 4218 ± 216 g), which were uniformly bred by Sichuan Animal Science Academy (Chengdu, China). All rabbits were fed individually in each cage under the same atmosphere, and each lactating rabbit nursed eight kits. The rabbit diet was supported by Sichuan Xinye Meilin Feed Co., Ltd., and consisted of Jiajie Meat Rabbit Compound Feed (Grade II). Rabbits could access water and feed freely. About 1.5 ~ 2.0 mL of colostrum (RC, *n* = 18) and RM (RM, *n* = 18) were collected from different lactation periods after parturition (the 1st day and 17th day). The samples were transported to the laboratory by dry ice and stored at −80 °C for analysis. Before we conducted the analysis, 1 mL of rabbit milk from each sample was removed, and 6 samples from RC or RM were well mixed in a single centrifuge tube to reduce individual variability. Hence, 3 pooled samples for RC (RC1, RC2, and RC3) and 3 pooled samples for RM (RM1, RM2, and RM3) were obtained and used to conduct the following proteomic and statistical analyses.

### 2.2 Nile red staining

We conducted Nile red staining according to the published method with some modifications (29). Fifty microliter of samples from RC or RM and the same volume of water were mixed in a centrifuge tube to dilute the samples. In a previous study, Nile Red, a fat-specific dye, was used to label the triglycerides in the core of milk fat globules in the samples (30). Briefly, Nile Red was added to the diluted samples individually to reach a final concentration of 0.012 g/L, and the mixtures were incubated in the dark at room temperature for 45 min. Meanwhile, low-melting-point agarose was prepared at a concentration of 5 g/L and stored in a water bath at 45 °C until use. After that, 5 μL of the sample was slowly mixed with 20 μL of low-melting-point agarose, and the mixture was then dropped onto the microscope slide. The coverslips were used to cover the drops to prevent the sample from drying. The staining graphs of each sample were captured using a fluorescence microscope (Olympus, Japan) under uniform parameters.

### 2.3 Isolation of MFGM proteins

The extraction of MFGM proteins in RC and RM was based on a previous study, with some modifications (31). Briefly, the cryopreserved samples of RC and RM were remelted at 4 °C. Then, a portion of the sample was pipetted into 1.5 mL centrifuge tubes and centrifuged at 3,000 *g* for 15 min at 4 °C to collect the upper fat layer. The fat layer was mixed with 10 times the volume of PBS (0.1 M) and centrifuged at 3,000 *g* for 10 min at 4 °C. The supernatant was removed, and the fat layer was retained. An equal volume of 0.2% SDS was added to the fat layer, mixed thoroughly, and then ultrasonicated for 1 min. Then, the mixture was centrifuged at 12,000 *g* for 10 min, and the supernatant (MFGM proteins) was pipetted to a new tube. The concentration of the extracted MFGM protein was determined by using the BCA protein concentration assay kit (Beyotime Biotech. Inc., China).

### 2.4 SDS-page

Ten micrograms of MFGM protein from RC or RM was mixed with the sample buffer and heated in boiling water for 5 min. The supernatant was obtained by centrifugation at 10,000 g for 20 min at 4 °C. Subsequently, a 12% SDS polyacrylamide gel was used to separate the protein at a constant voltage of 80 V for 30 min, followed by electrophoresis at 120 V for 2 h. The gel was stained with Coomassie Brilliant Blue (CBB) for 60 min to visualize the protein bands, then decolorized with methanol: glacial acetic acid (4:1) solution for 1 h to obtain the separation images. The iBright imaging system (Thermo Fisher Scientific, United States) was used to capture the images.

### 2.5 Digestion of MFGM proteins

The same amount of MFGM proteins from RC or RM was subjected to FASP enzymatic hydrolysis to facilitate further analysis. Then, the DTT reagent was added to each sample to reach a final concentration of 100 mM, and the samples were heated in a boiling water bath for 5 min. After the samples were cooled to room temperature, 200 μL of UA buffer (8 M urea, 150 mM Tris–HCl, pH 8.0) was added and mixed well. Then, the mixtures were transferred to the 10 KD ultrafiltration centrifuge tube and centrifuged at 12,000 *g* for 15 min. After that, 200 μL of UA buffer was added to the tube, the tube was centrifuged at 12,000 *g* for 15 min, and then the filtrate was discarded. Subsequently, 100 μL of IAA (50 mM IAA in UA) was added to the sample tubes, and the tubes were oscillated at 600 rpm for 1 min. The samples were incubated at room temperature in the dark for 30 min and centrifuged at 12,000 *g* for 10 min. After that, 100 μL of UA buffer was added and centrifuged at 12,000 *g* for 10 min, which was repeated twice. Then, 100 μL of NH_4_HCO_3_ buffer was added to each tube, and the tubes were centrifuged at 14,000 *g* for 10 min, which was repeated twice. Next, 40 μL of trypsin buffer (6 μg trypsin in 40 μL NH_4_HCO_3_ buffer) was added and shaken at 600 rpm for 1 min, followed by an incubation at 37 °C for 16–18 h. The mixture was centrifuged at 12,000 *g* for 10 min, and the filtrate was collected in the new collection tubes. An appropriate amount of 0.1% TFA solution was added to the filtrate, and the peptide segments after enzymatic hydrolysis were desalted using a C18 cartridge and vacuum freeze-dried. Finally, the dried peptides were redissolved with 0.1% FA, and the concentration was determined.

### 2.6 Mass spectrometry assay for data independent acquisition (DIA)

For the first part, the preprocessed peptides in RC and RM were administered for chromatographic separation using the Vanquish Neo UHPLC system (Thermo Scientific). With respect to this, buffer A was a 0.1% formic acid aqueous solution, and buffer B was a 0.1% formic acid acetonitrile aqueous solution (acetonitrile 80%). The chromatographic column was balanced with 96% buffer A. The sample was individually injected into the Trap Column (PepMap Neo 5 μm C18 300 μm × 5 mm, Thermo Scientific) and separated by the chromatographic analysis column (μPAC Neo High Throughput column, Thermo Scientific). The separation method was conducted as follows: 0–0.1 min, buffer B 4–6%; 0.1–1.1 min, buffer B 6–12%; 1.1–4.3 min, buffer B 12–22.5%; 4.3–6.1 min, buffer B 22.5–45%; 6.1–8 min, buffer B was maintained at 99%. For the second part, after the peptide separation, data-independent acquisition (DIA) mass spectrometry was performed on an Orbitrap Astral mass spectrometer (Thermo Scientific). The mass spectrometer was operated in positive ion mode, and the total analysis time was 8 min. The parameters were set as follows: electrospray voltage: 2.2 kV, parent ion scanning range: 380–980 m/z, primary mass spectrometry resolution: 2,40,000, automatic gain control (AGC) target: 500%, primary maximum injection time (Maximum IT): 3 ms. The resolution of the secondary mass spectrometry was 80,000. The AGC target was 500%, and the secondary maximum IT was 3 ms. The other parameters were set as follows: RF-lens: 40%, MS2 Activation Type: HCD, Isolation window: 2 Th, Normalized collision energy: 25%, cycle time: 0.6.

### 2.7 Database search and bioinformatics analysis

The DIA raw data for each pooled sample were combined and searched using the DIA-NN software for identification and quantitation analysis. The parameter set of DIA-NN was as follows: Enzyme: Trypsin; Max missed cleavages: 1; Fixed modifications: Carbamidomethyl (C); Variable modifications: Oxidation (M), Acetyl (Protein N-term); Database: uniprot-*Oryctolagus cuniculus* (Rabbit) [9986]-43525-20231109.fasta; Database pattern: Target-Reverse; PSM (Peptide-Spectral Matching) FDR: 0.01; Protein FDR: 0.01. All reported data were based on 99% confidence in protein identification as determined by false discovery rate (FDR) ≤ 1%. For the identified results, 31,285 precursors and 4,018 protein groups were obtained.

To ensure the validity and accuracy of subsequent bioinformatics and statistical analyses, we first screen the sample experimental data in the protein identification table to retain only proteins for which at least 50% of the non-null data are available. Then, we fill in the remaining null values and conduct a statistical analysis. In protein significant difference analysis, Student’s *t*-test and the fold change (FC, the ratio of the mean value of expression between two groups) method were used jointly. Proteins that met the screening criteria of FC (RC/RM) > 1.5 (or <1/1.5) and *p*-value < 0.05 were considered as significant DEMPs.

### 2.8 Advanced analysis of proteomic data

The DEMPs identified in RC and RM were uploaded to the online software for Gene Ontology (GO) annotation^1^ and Kyoto Encyclopedia of Genes and Genomes (KEGG) pathway analysis^2^, and the annotation results were plotted. The whole proteins were used as the background dataset, and the annotation results were analyzed for functional enrichment using Fisher’s exact test algorithm. The screened DEMPs were analyzed for protein functional interaction network PFIN, which was integrated into the network by combining the protein–protein interaction relationships from the STRING database^3^ and the relationships between pathways and proteins. The top 50 vertices were selected using the PageRank algorithm to construct the network. Principal Component Analysis (PCA) was used to verify group clustering. The median coefficient of variation (CV) of peptide intensity across three technical replicates was <20%, meeting DIA proteomics data quality standards. Additionally, the R language limma package was used to construct a linear model, which effectively eliminates batch effects and intra-group variation.

### 2.9 Parallel reaction monitoring (PRM) detection

To verify the differential expression of MFGM proteins identified in RC and RM, several proteins with large differences in proteomic data (Fold change > 2 and *p*-value < 0.05) were selected for PRM detection. The peptide information suitable for PRM analysis was imported into Xcalibur™ software for PRM calibration. One microgram peptide mixture per sample was separated by an EASY-nLC 1,200 HPLC system. The mobile phase A was 0.1% formic acid, and the mobile phase B was 84% acetonitrile plus 0.1% formic acid. The column was equilibrated with 95% mobile phase A. Then, the samples were loaded onto a collection column via an autosampler and separated on a Thermo Scientific EASY column (75 μm × 200 mm, 3 μm- c18) in a gradient at a flow rate of 5 μL/min. Samples were separated as follows: 5–10% buffer B for 0–2 min, 10–30% buffer B for 2–45 min, 30–100% buffer B for 45–55 min, and 100% buffer B for 55–60 min.

The separated samples were analyzed by PRM mass spectrometry using a Q Exactive™ HF mass spectrometer (Thermo Scientific). The mass spectrometer was operated in positive ion mode for 30 min, and the scanning range was 300–1,800 m/z. The mass spectral resolution was 60,000 (m/z 200), and the automatic gain control (AGC) target was 3e6. The maximum ion injection time (IT) was set to 200 ms. After each full MS, PRM scans were collected according to the inclusion table. The main parameters were set as follows: mass spectrometry resolution: 30000 (@m/z 200), AGC target: 3e6, maximum IT: 120 ms, MS2 activation type: HCD, isolation window: 1.6 Th, normalized collision energy: 27 eV. The Skyline software (version 3.5.0) was used to analyze the original PRM file.

### 2.10 Statistical analysis

All data were presented as means ± standard deviation (SD), and the Student’s *t*-test was used to test the significant differences between the two groups. Statistical analyses were performed using GraphPad Prism (GraphPad Software, Santiago, United States), and a *p*-value <0.05 indicated a significant difference.

## 3 Results

### 3.1 Depiction of the protein profile of milk fat globule membrane (MFGM) in rabbit colostrum (RC) and mature milk (RM)

**Figure 1:**
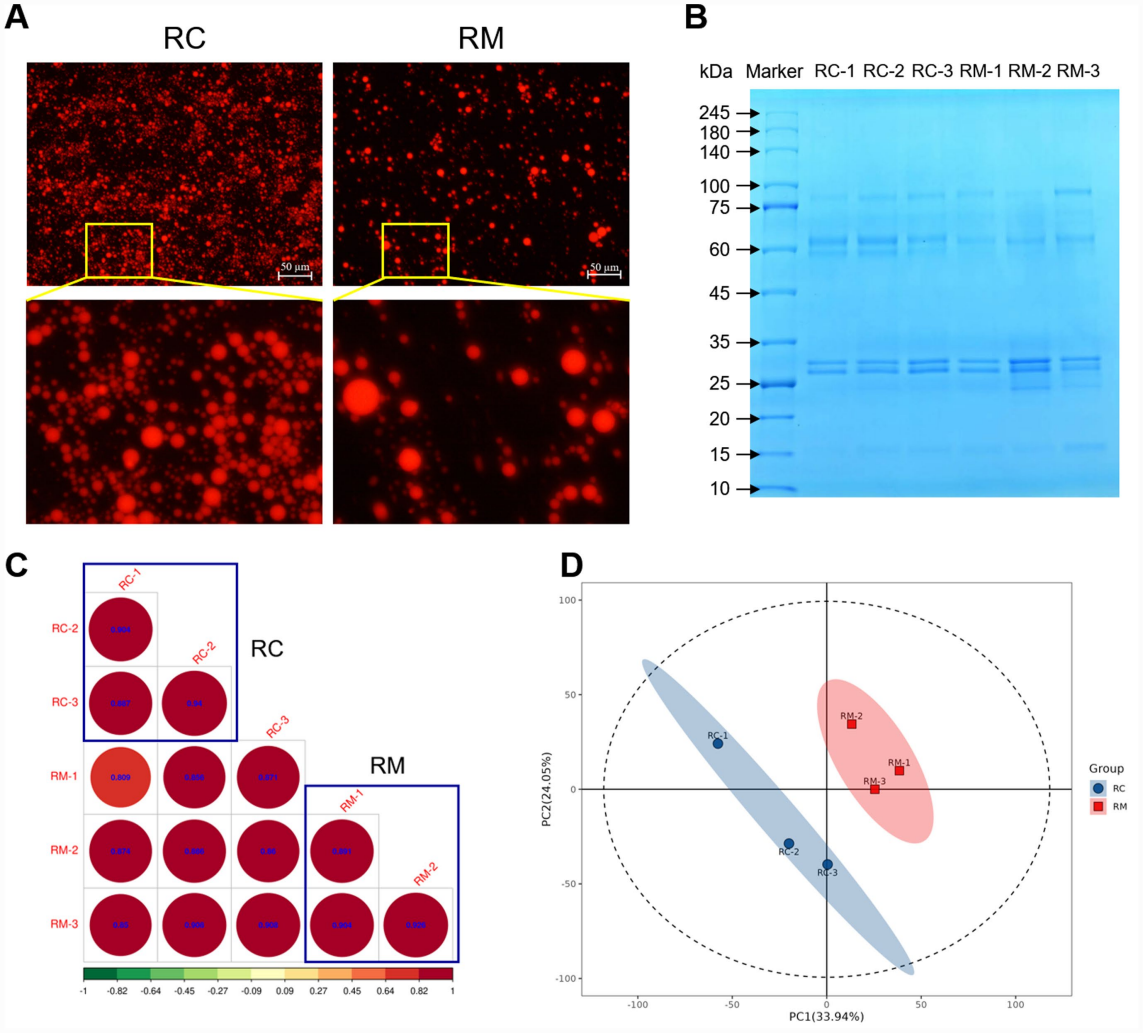
Characteristics of milk fat globule membrane (MFGM) and MFGM proteins in rabbit colostrum (RC) and mature milk (RM). **(A)** Representative images of Nile red staining in RC and RM. **(B)** Distribution diagram of MFGM proteins in RC and RM detected by SDS-PAGE. **(C)** Sample correlation analysis of samples. **(D)** Principal component analysis (PCA) of samples. Three pooled replicates existed in each group (*n* = 3).

First, we collected RC and RM from healthy lactating rabbits at days 1 and 17 post-partum between 8:00 and 10:00 a.m. daily (Supplementary Figure S1). Then, we observed the milk fat globule by using Nile red staining (29). Generally, the quantity and volume size of milk fat globules implied a diversity in RC and RM (Figure 1A). Meanwhile, we isolated the MFGM proteins from RC and RM. We analyzed their qualities and protein distribution for the first time. The majority of the purified MFGM proteins had molecular weights (MW) ranging from 25 to 100 kDa, which helps define the MW characteristics of rabbit MFGM proteins (Figure 1B). Briefly, we identified a total of 31,285 peptide fragments across the samples, and 3,548 proteins were ultimately mapped in them (Supplementary Tables S1, S2).

Next, we conducted the correlation analysis and found that the sample correlation coefficient exceeded 0.88, showing a high correlation in RC and RM (Figure 1C). The principal component analysis (PCA) result displayed an obvious disparity in RC and RM, with 33.94% of principal component (PC) 1 and 24.05% of PC2 (Figure 1D). Additionally, the predominant lactoproteins, casein and whey protein for instance, were found both in RC and RM, which was consistent with the previous studies (25, 32). To summarize, we compared the morphology of MFGM in RC and RM, then extracted MFGM proteins from them. For the first time, the expression profile of rabbit MFGM proteins was depicted in the present study.

### 3.2 Identification of differentially expressed MFGM proteins (DEMPs) in RC and RM

**Figure 2:**
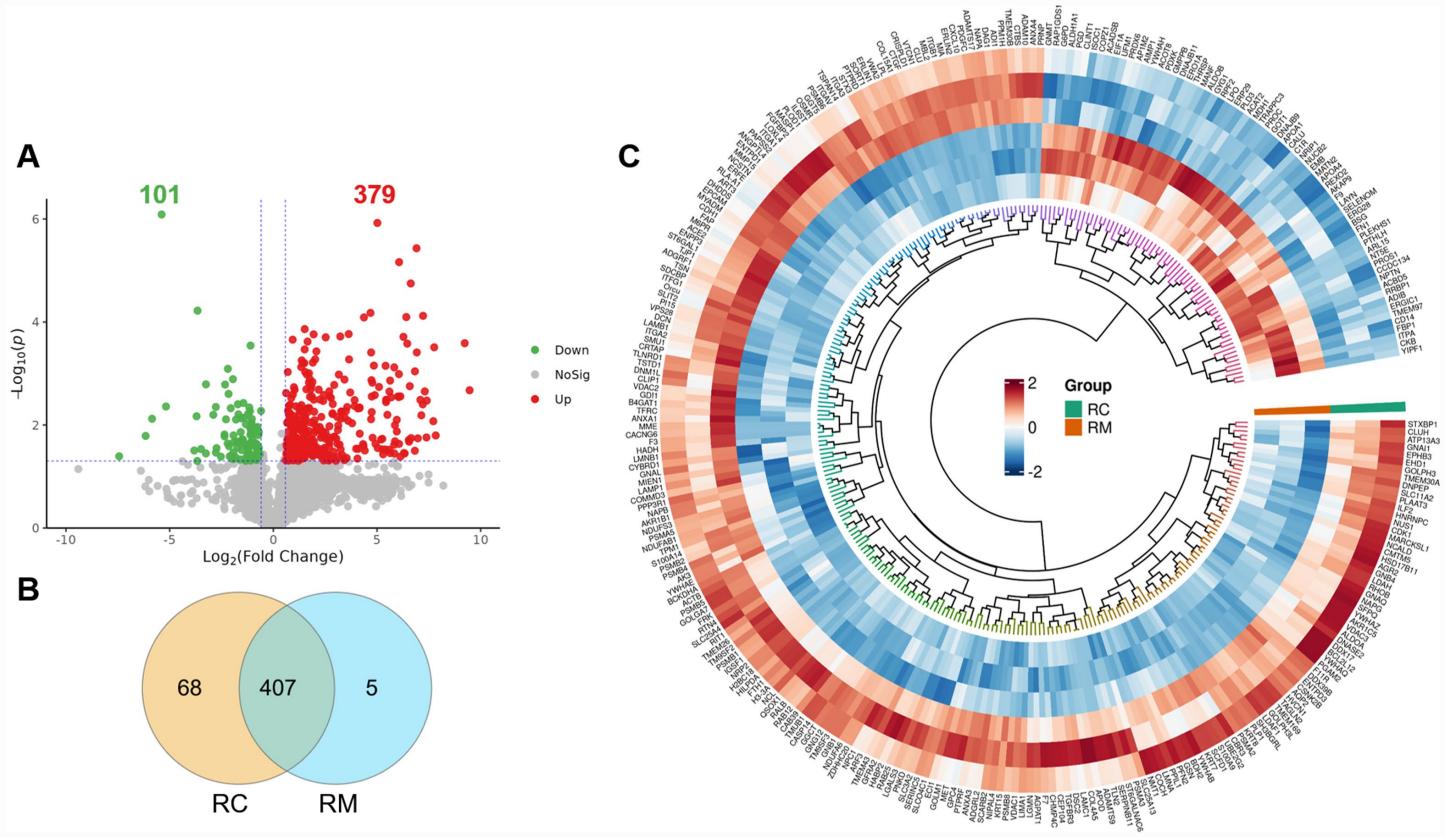
Identification of differentially expressed MFGM proteins (DEMPs) in RC and RM. **(A)** Volcano plot of DEMPs in RC and RM. **(B)** Venn map exhibition of DEMPs uniquely detected in RC or RM. **(C)** Circle heatmap exhibition of partial DEMPs in RC and RM. Three pooled replicates existed in each group (*n* = 3).

In the present study, to determine the DEMPs between RC and RM, we defined the statistically significant threshold value as FC (RC/RM) > 1.5 (or <1/1.5) and *p*-value < 0.05, and the uniquely expressed proteins were also defined as the DEMPs. A total of 480 DEMPs were acquired from different phases of rabbit lactation, including 379 up-regulated DEMPs and 101 down-regulated DEMPs in RC compared to RM (Figure 2A and Supplementary Table S3). Among them, 68 and 5 unique proteins were detected in RC and RM, respectively, with 407 overlapping DEMPs (Figure 2B). We also showed partial DEMPs in a circle heatmap (Figure 2C).

We further displayed the top 15 unique or up/down-regulated DEMPs in RC and/or RM (Table 1). Accordingly, proteasome assembly chaperone 2 (PAC2), FAT atypical cadherin 2 (FAT2), Thrombomodulin (THBD), Myelin proteolipid protein (PLP1), Inhibin subunit beta B (INHBB), Thioredoxin-related transmembrane protein 3, Integrin alpha-2 domain-containing protein, GPI-anchor transamidase (PIGK), etc., were uniquely detected in RC (Table 1). Moreover, five DEMPs, including Uracil phosphoribosyl transferase homolog (UPRT), Bet1 Golgi vesicular membrane trafficking protein (BET1), Integral membrane protein 2 (ITM2B), Solute carrier family 25 member 17 (SLC25A17) and Inosine triphosphate pyrophosphatase (ITPA), were only detected in RM (Table 1), most of which were determined as the MFGM proteins for the first time.

**Table 1:** Top 15 unique or up/down-regulated DEMPs in RC and/or RM.

Accession	Protein name	*P*-value	Fold change	Change
A0A5F9CFJ4	Proteasome assembly chaperone 2	1.19E-06		Unique in RC
G1T901	ADAM metallopeptidase with thrombospondin type 1 motif 17	3.71E-06		Unique in RC
A0A5F9CAH0	Structural maintenance of chromosomes protein 3	6.88E-06		Unique in RC
A0A5F9C490	FAT atypical cadherin 2	1.79E-05		Unique in RC
Q8HZ48	Thrombomodulin	6.65E-05		Unique in RC
P47789	Myelin proteolipid protein	7.59E-05		Unique in RC
G1SN71	Inhibin subunit beta B	8.02E-05		Unique in RC
A0A5F9DTI5	Stimulated by retinoic acid 6	1.94E-04		Unique in RC
G1TVQ0	MIA SH3 domain containing	2.58E-04		Unique in RC
G1SMZ8	Thioredoxin related transmembrane protein 3	2.63E-04		Unique in RC
A0A5F9C1R9	von Willebrand factor A domain containing 2	3.10E-04		Unique in RC
G1TR44	Integrin alpha-2 domain-containing protein	3.90E-04		Unique in RC
G1SI76	Large ribosomal subunit protein mL43	7.03E-04		Unique in RC
G1SPG6	GPI-anchor transamidase	7.09E-04		Unique in RC
G1U3I5	Heterogeneous nuclear ribonucleoprotein L	7.61E-04		Unique in RC
A0A5F9DVV0	Uracil phosphoribosyl transferase homolog	8.18E-07		Unique in RM
A0A5F9D3Z6	Bet1 Golgi vesicular membrane trafficking protein	4.38E-03		Unique in RM
A0A5F9CJB1	Integral membrane protein 2	7.56E-03		Unique in RM
A0A5F9CMR7	Solute carrier family 25 member 17	1.63E-02		Unique in RM
A0A5F9CYL3	Inosine triphosphate pyrophosphatase	4.05E-02		Unique in RM
A0A5F9CSK7	Aquaporin-5	8.15E-05	20.7019	Up
A0A5F9CZT7	RRM domain-containing protein	1.37E-04	2.8566	Up
G1TDA1	Platelet-derived growth factor C	1.72E-04	12.4317	Up
G1SS18	Keratin 8	1.74E-04	3.8472	Up
G1TIT1	Erlin	1.91E-04	9.3709	Up
G1T2A1	Voltage-gated hydrogen channel 1	1.98E-04	5.7607	Up
G1T2L1	Proteasome subunit alpha type	2.20E-04	1.9123	Up
A0A5F9DA41	Insulin-like growth factor-binding protein 2	2.40E-04	3.1412	Up
G1TAY6	Keratin 15	3.41E-04	2.8531	Up
G1T6L5	SH3 domain binding glutamate-rich protein-like	3.72E-04	4.2214	Up
A0A5F9CHA2	Galactocerebrosidase	3.90E-04	26.2832	Up
A0A5F9C3C4	Integrin beta	5.30E-04	4.0479	Up
A0A5F9CZS9	Collagen type XV alpha 1 chain	5.33E-04	12.5887	Up
G1SF09	NIPA-like domain containing 4	5.58E-04	2.5845	Up
G1SRX2	116 kDa U5 small nuclear ribonucleoprotein component	6.13E-04	3.7458	Up
G1SY10	SPARC (osteonectin), cwcv., and kazal-like domains proteoglycan 1	6.03E-05	0.0795	Down
Q28661	Vitamin K-dependent protein C (Fragment)	2.87E-04	0.4668	Down
A0A5F9C853	Acyl-CoA-binding domain-containing protein 5	8.11E-04	0.2189	Down
G1TBH3	DnaJ heat shock protein family (Hsp40) member B9	1.29E-03	0.2594	Down
A0A5F9DSX4	Collagen type V alpha 2 chain	1.63E-03	0.1051	Down
A0A5F9DH54	Ubiquitin-fold modifier 1	1.64E-03	0.1994	Down
A0A5F9D7H4	Heparanase	2.49E-03	0.2242	Down
G1T332	Aspartate aminotransferase	3.74E-03	0.3680	Down
A0A5F9CB01	Neuroplastin	3.95E-03	0.3058	Down
G1T958	EXPERA domain-containing protein	4.47E-03	0.1462	Down
P13280	Glycogenin-1	4.57E-03	0.4480	Down
A0A5F9D8X3	Matrilin 2	4.72E-03	0.2408	Down
G1T0H7	Trafficking protein particle complex subunit	5.32E-03	0.6637	Down
Q8MI17	Aldehyde dehydrogenase 1A1	5.42E-03	0.4590	Down
A0A5F9DGZ3	Endoplasmic reticulum-Golgi intermediate compartment protein	6.13E-03	0.4281	Down

The main up-regulated DEMPs in RC compared to RM were Aquaporin-5 (AQP5), RRM domain-containing protein, Platelet-derived growth factor C (PDGF-C), Keratin 8, etc. Furthermore, the down-regulated DEMPs in RC, which were highly expressed in RM, mainly contained SPARC (osteonectin), CWcv., and kazal-like domains proteoglycan 1 (SPOCK1), vitamin K-dependent protein C (Fragment), Acyl-CoA-binding domain-containing protein 5, Heparanase, Aspartate aminotransferase, etc. (Table 1). These results resolved the differences in MFGM proteins between RC and RM, and might imply the specific nutrient requirements for neonatal rabbits from maternal milk after delivery.

### 3.3 GO advanced analysis of DEMPs in RC and RM

**Figure 3:**
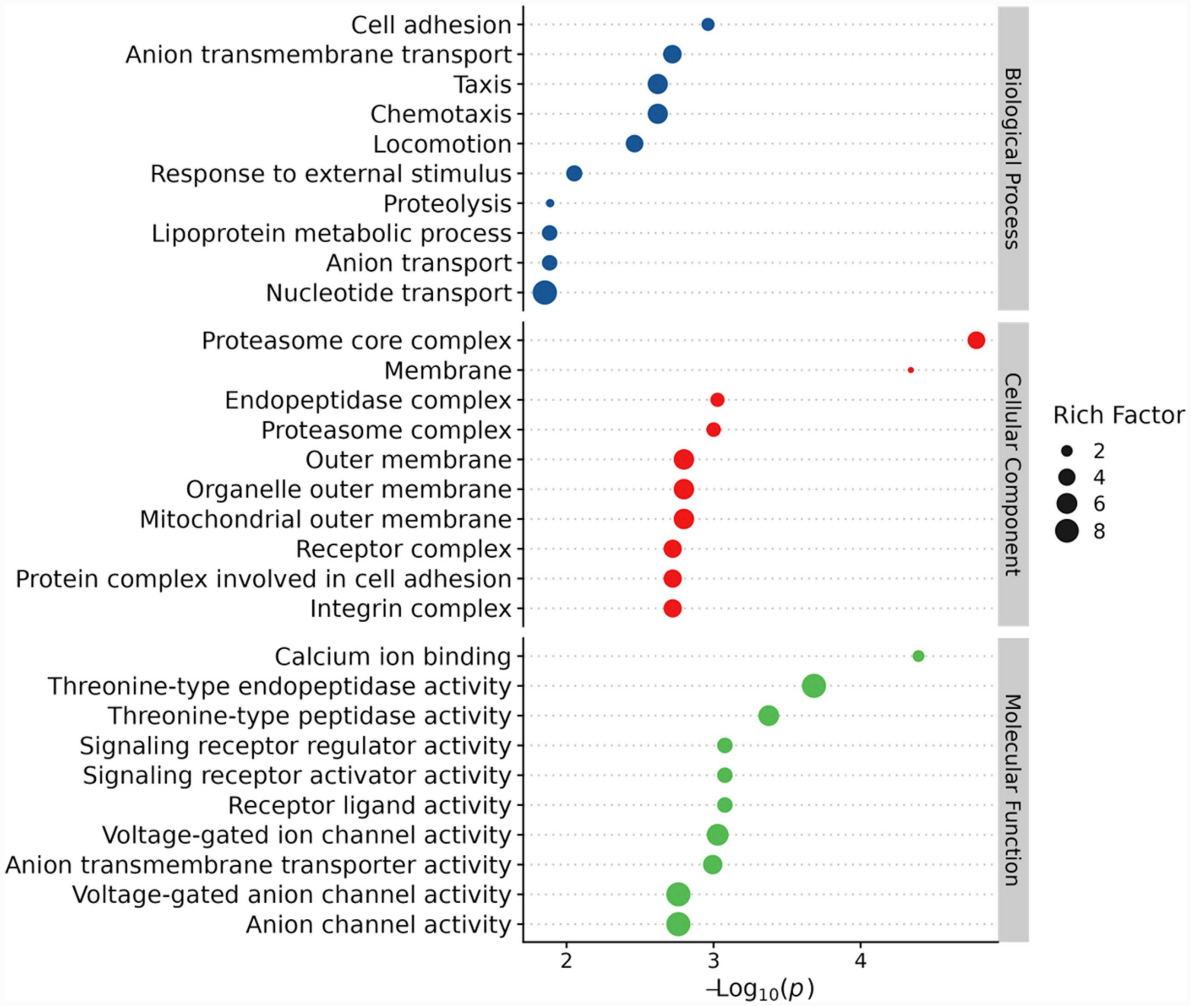
GO annotation of DEMPs in RC and RM.

The GO analysis results showed that 480 DEMPs were classified into biological processes (BP), cellular components (CC), and molecular functions (MF), with 38, 16, and 49 items, respectively, enriched in each sub-component (*p* < 0.05) (Supplementary Table S4). We listed and further analyzed the top 10 items in BP, CC and MF (Figure 3). As shown in Figure 3, items mainly gathered in BP were involved in cell adhesion, transportation (anion transmembrane transport, anion transport, and nucleotide transport), chemotaxis and stress response (taxis, chemotaxis, response to external stimulus, and locomotion), and protein metabolism (proteolysis and lipoprotein metabolic process). The DEMPs were primarily enriched in proteolysis (32), cell adhesion (14), and ion transport (14) in the BP subgroup (Supplementary Table S4). Similar to the results obtained from the pig (25), human, and donkey (11), the CC subgroup was mainly related to membrane structure (membrane, outer membrane, organelle outer membrane) and complex formation (proteasome core complex, endopeptidase complex, proteasome complex). Meanwhile, the most common MF was calcium ion binding, and catalytic activities, the latter included threonine-type endopeptidase activity, threonine-type peptidase activity, signaling receptor regulator activity, signaling receptor activator activity, etc. (Figure 3).

To further explore the functional differences of MFGM proteins in various stages of rabbit lactation, 379 up-regulated DEMPs and 101 down-regulated DEMPs were individually subjected to the GO analysis. Similar to the above results, DEMPs were separated into BP, CC, and MF subgroups (Supplementary Tables S5, S6). In the top 10 items enriched in BP, up-regulated DEMPs were mainly included in cell adhesion, proteolysis, and various transportation (ion transport, anion transport, and nucleotide transport). However, most down-regulated DEMPs enriched in BP were involved in chemotaxis (taxis and chemotaxis) and metabolism (vitamin B6 metabolic process and nucleotide-sugar metabolic process). Meanwhile, up-regulated DEMPs enriched in the CC subgroup were primarily associated with membrane structure and various complexes (e.g., proteasome core complex and endopeptidase complex), whereas down-regulated DEMPs were largely associated with membrane structure. Finally, up-regulated DEMPs enriched in the MF subgroup mostly participated in channel and catalytic activities, whereas down-regulated DEMPs mainly took part in ligand and receptor activities (Supplementary Figure S2). These results demonstrated that up- or down-regulated DEMPs in RC and RM were involved in different life processes, implying the specific functions of MFGMs across various phases of rabbit lactation.

### 3.4 KEGG pathway analysis of DEMPs in RC and RM

**Figure 4:**
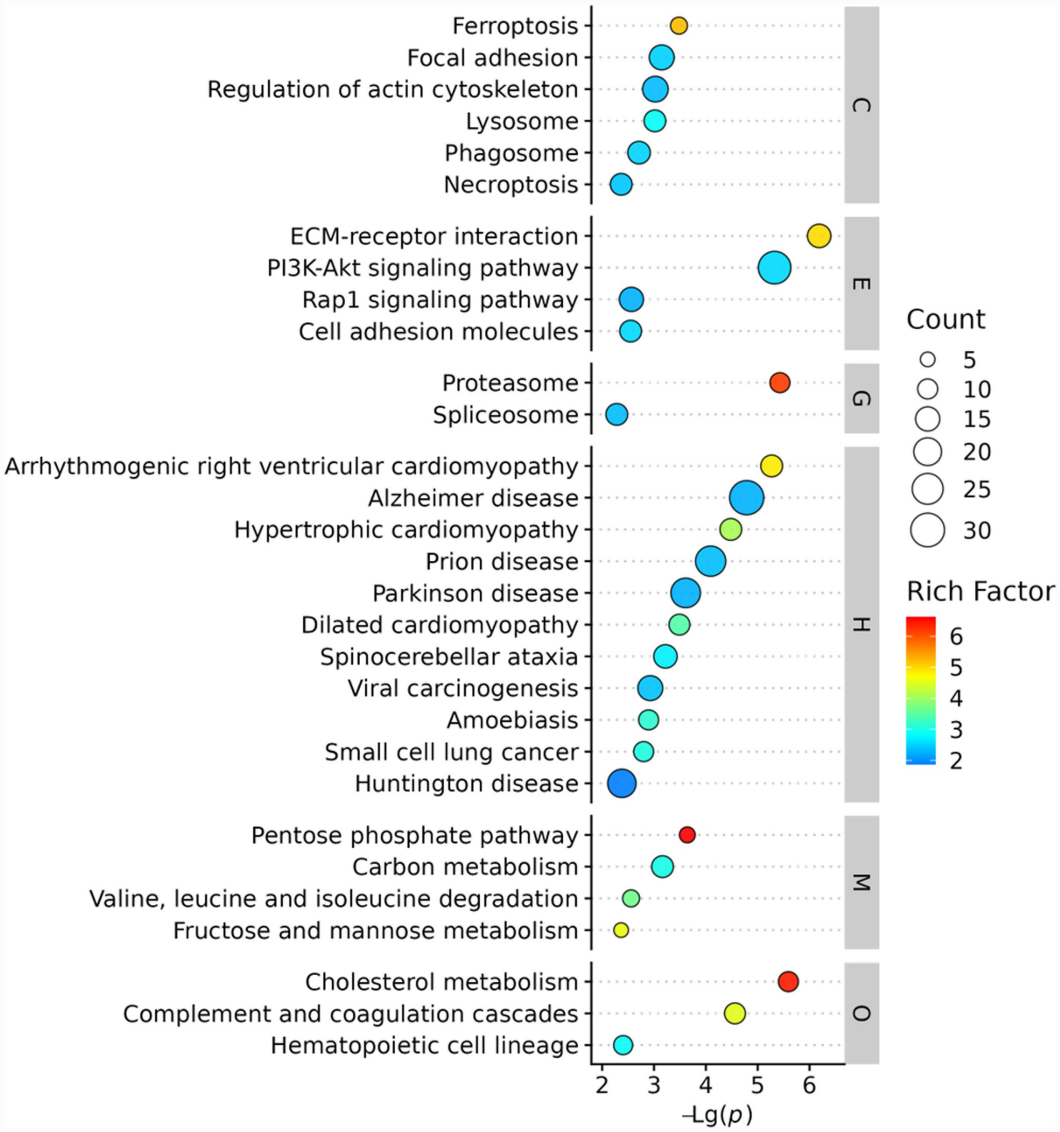
KEGG pathway analysis of DEMPs in RC and RM. (C) Cellular processes; (E) environmental information processing; (G) genetic information processing; (H) human diseases; (M) metabolism; (O) organismal systems.

We performed KEGG pathway analysis of the above DEMPs. A total of 56 pathways were significantly enriched (*p* < 0.05), which were categorized into five classes, including Human Diseases (20/56), Metabolism (14/56), Cellular Processes (9/56), Environmental Information Processing (6/56), Organismal Systems (5/56), and Genetic Information Processing (2/56) (Supplementary Table S7). In the top 30 pathways exhibited in Figure 4, most pathways were classified in Human Diseases (11/30), such as Alzheimer’s disease and hypertrophic cardiomyopathy. Pathways enriched in Cellular Processes (6/30) were mainly related to programmed cell death (PCD) and autophagy (Ferroptosis, Necroptosis, Phagosome, and Lysosome). The remaining pathways belonged to Environmental Information Processing (4/30) and Metabolism (4/30); the former emphasized key aspects of life processes, including the PI3K-Akt signaling pathway, Rap1 signaling pathway, and ECM-receptor interaction, and the latter highlighted carbon and amino acid metabolism. Additionally, proteasome and cholesterol metabolism were, respectively, enriched in the Genetic Information Processing and Organismal Systems (Figure 4).

Consistently, we conducted the independent KEGG pathway analysis for the up- and down-regulated DEMPs. Similarly to the above results, the up-regulated DEMPs were mainly enriched in Human Diseases (21/55), implying they dominated the enrichment of KEGG pathway (*p* < 0.05) (Supplementary Figure S3A and Supplementary Table S8). Interestingly, the down-regulated DEMPs were mostly classified in Metabolism (18/28) (*p* < 0.05), which were associated with carbon metabolism, amino acid and vitamin metabolism, and lipid metabolism (Supplementary Figure S3B and Supplementary Table S9). These results indicated that an obvious discrepancy of MFGM proteins existed between RC and RM, the former of which was inclined to human diseases, and the latter of which was apt to metabolism. In summary, the up-regulated DEMPs in RC were mainly enriched in human disease-related pathways, and some key processes, such as ferroptosis, lysosome, and molecular pathways (i.e., PI3K and ECM-receptor interaction), were enriched likewise. Conversely, down-regulated DEMPs in RC, which were highly expressed in RM, were mainly classified in metabolism, highlighting the difference between RC and RM.

### 3.5 Protein–protein interaction (PPI) network analysis of DEMPs

**Figure 5:**
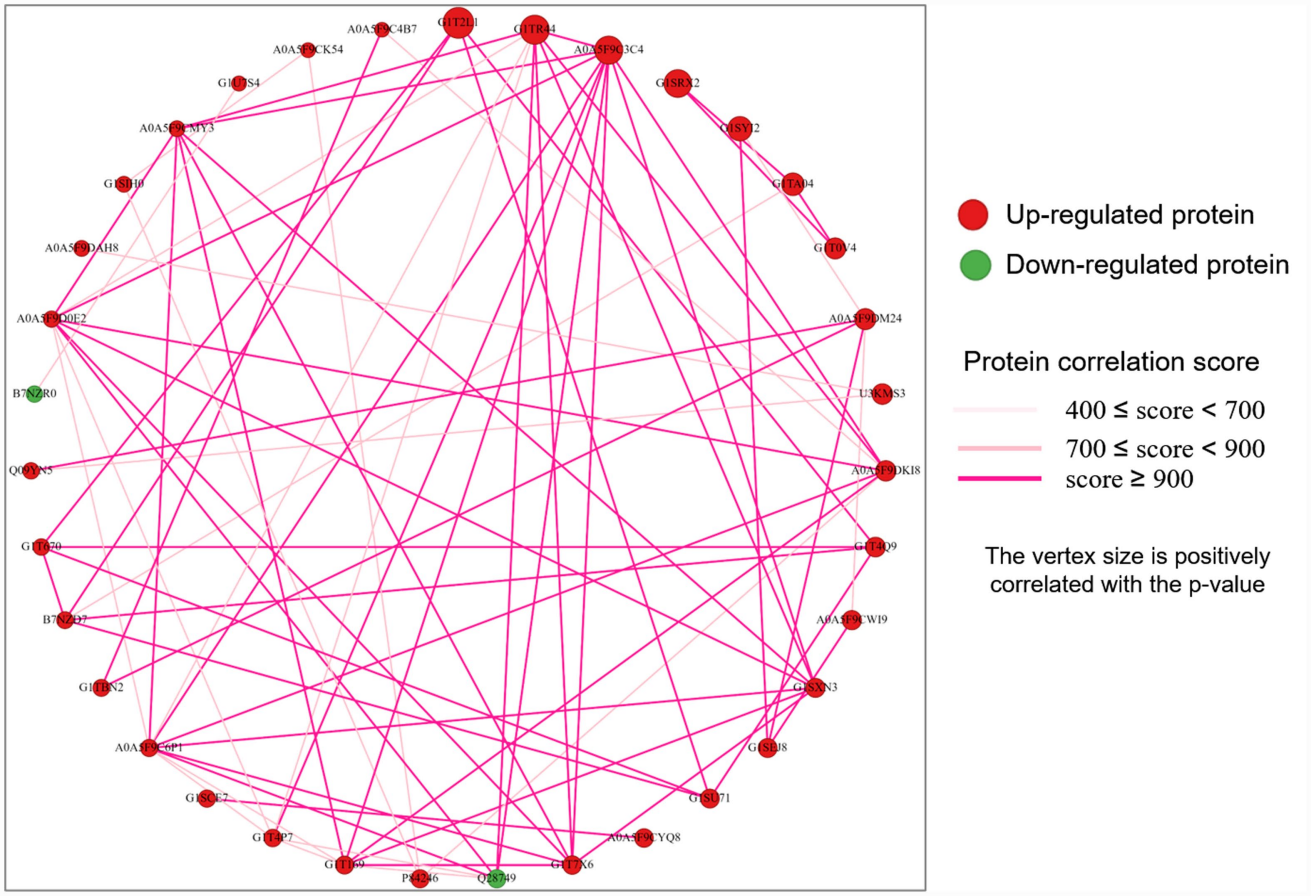
Protein–protein interaction (PPI) network analysis of the top 35 DEMPs in RC and RM.

Next, we conducted PPI network analysis of DEMPs using the STRING database. The results showed that a total of 256 DEMPs were involved in the interactions, among them, 209 were up-regulated, and 47 were down-regulated DEMPs in RC compared to RM (Supplementary Figure S4). Furthermore, using the PageRank algorithm, the top 50 vertices were selected to construct the network, and 35 DEMPs were enriched, yielding 74 interactions. Accordingly, 33 DEMPs were up-regulated in RC, Integrin beta (ITGB1, 10 interactions), Integrin subunit alpha 2 (ITGA2, 10 interactions), Integrin alpha-2 domain-containing protein (9 interactions), and Integrin subunit alpha 1 (ITGA1, 8 interactions), for instance. Only Fibronectin (FN1, 6 interactions) and Glucose-6-phosphate 1-dehydrogenase (G6PD, 1 interaction) were down-regulated DEMPs (Figure 5). Considering its biological roles in cells and specific expression pattern in rabbit milk, we speculated that FN1 might play a particular role in RM, such as colonization of enteric microorganisms; however, further exploration is required to solve this mystery.

### 3.6 PRM analysis of DEMPs in RC and RM

The PRM analysis has been widely used to verify the reliability of proteomic data (25, 26). Hence, to validate the reliability of our data, 10 representative DEMPs (e.g., DAG1, LPL, VDAC1, ANGPTL4, PSMA5, PDGFC, ITGB1, MIA, GOT1, and FN1) with distinct discrepancies were chosen, and conducted PRM analysis. Consistent with our expectations, the PRM results were aligned with the DIA quantitative data (Table 2), demonstrating the repeatability of our proteomic data.

**Table 2:** Parallel reaction monitoring (PRM) analysis of selected DEMPs detected in RC and RM.

Accession	Gene name	Protein	PRM data		DIA quantitative data		Change
			Fold change	*P*-value	Fold change	*P*-value	
Q28685	DAG1	Dystroglycan 1	11.6798	4.67E-02	3.6259	2.80E-03	Up
D5FIT0	LPL	Lipoprotein lipase	3.6591	1.12E-02	2.8276	6.00E-04	Up
Q9TT15	VDAC1	Voltage-dependent anion-selective channel protein 1	3.8014	3.55E-02	3.2960	1.00E-03	Up
G1TBZ2	ANGPTL4	Angiopoietin-related protein 4	2.9467	4.54E-02	2.4234	2.16E-02	Up
G1T670	PSMA5	Proteasome subunit alpha type	2.7502	4.64E-02	2.2676	2.35E-02	Up
G1TDA1	PDGFC	Platelet-derived growth factor C	25.1610	9.30E-03	12.4317	2.00E-04	Up
A0A5F9C3C4	ITGB1	Integrin beta	8.0139	6.00E-04	4.0479	5.00E-04	Up
G1TVQ0	MIA	MIA SH3 domain containing	54.2329	1.00E-04	601.3608	3.00E-04	Up
G1T332	GOT1	Aspartate aminotransferase	0.3717	5.00E-03	0.3680	3.70E-03	Down
Q28749	FN1	Fibronectin	0.3891	3.10E-02	0.3359	1.37E-02	Down

## 4 Discussion

Prevalent proteomic quantitative technologies have been broadly used to identify MFGM proteins encompassing TMT (21), iTRAQ (22, 33), DIA (23) and LFQP (10), and mass milk MFGM proteins in different phases of lactation have been obtained from humans (33), bovine (32), goat (34, 35), pig (25), and donkey (26). Compared with the recent report from Huang et al. (36), the present study introduces DIA quantitative proteomics to depict the expression profiles of MFGM proteins in RC and RM for the first time.

Accordingly, we obtained 3,548 proteins from rabbit milk, including some classic MFGM proteins, such as Perilipin 2 (PLIN2), Xanthine dehydrogenase/oxidase (XDH/XO), Cell death inducing DFFA-like effector (CIDE), Butyrophilin subfamily 1 member A1 (BTN1A1), CD36, Mucin, Apolipoprotein, etc., most of which have been acquired from the mammals (Supplementary Table S2) (10, 25, 26, 32–34), implying the functional evolutionary conservation of MFGM proteins. Briefly, PLIN2, XDH/XO, CIDE, and BTN1A1 are associated with the synthesis and secretion of neutral lipids by a unique apocrine mechanism in milk-secreting epithelial cells of the mammary gland (37). Specifically, although members of the Perilipin share a similar domain organization, PLIN2, an important regulator of lipid metabolism that exhibits the opposite function to PLIN1 and PLIN5, appears to package the lipid droplets and protect them from lipolysis (38). Furthermore, CD36, a widely expressed scavenger receptor, is reported to be involved in various lipid metabolism diseases (e.g., cancer) by mediating lipid uptake, molecular adhesion, and inflammation (39). Mucin, a highly glycosylated protein in both human and donkey colostrum and RM (11, 40, 41), plays a crucial role in promoting the intestinal equilibrium of infants. Another glycoprotein identified in donkey colostrum (41) and pig milk (25), apolipoprotein, works as the cofactor of lipid metabolic enzymes, which can transport and redistribute the plasma lipid, as well as maintain the structure of lipoprotein, to regulate various physiological processes, such as cardiovascular disease (42). Those results suggested the similarity of classical MFGM proteins between rabbits and mammals.

To comprehensively cognize the characteristics of rabbit milk, we identified 480 DEMPs from RC and mature milk, encompassing 379 up-regulated and 101 down-regulated MFGM proteins in colostrum (Supplementary Table S3). Notably, most DEMPs identified in this study were not similar with that from Huang et al. report (36), emphasizing the novelty of this study. The fundamental reason for this difference lies in the various research subjects [whole milk for Huang et al. (36); only MFGM proteins in the present study]. For example, in the top 15 unique DEMPs between RC and RM in this study (Table 1), only five proteins were presented in their results (36). Among the unique DEMPs in RC (Table 1 and Supplementary Table S3), PAC2 exhibited distinct biological properties. The biological function of proteasome is choreographed complicating processes to adjust cellular protein degradation, thereby orchestrating many life processes including transcription, protein trafficking, cell death and proliferation (43). Well-balanced proteasome assembly is fundamental for natural degradation of abandoned protein, which is tightly associated with PAC2, encoded by the *PSMG2* gene (43). Emerging evidence suggests that PAC2 plays a pivotal role in regulating the proteasome-autophagy balance (44), and codon mutation or knockdown of the *PSMG2* gene might contribute to severe anti-inflammatory diseases (45). These findings imply the essential function of PAC2 in sustaining normal cellular processes in newborn rabbits after suckling colostrum. Previous studies have revealed the existence of Integrin in human milk (11) and bovine milk (33), with the high glycosylation in donkey colostrum (41), which were engaged in the phagosome signaling pathway. Consistent with this, excepting Integrin alpha-2 domain-containing protein, which was only detected in RC, six Integrin-related proteins, i.e., Integrin beta, Integrin alpha FG-GAP repeat containing 1, Integrin subunit alpha 1, Integrin subunit alpha 2, Integrin subunit alpha V, and Integrin subunit alpha 3, were up-regulated in RC compared to RM (Supplementary Table S3), indicating their special roles in rabbit milk. As the main cell-adhesion transmembrane receptors, intensive researches have demonstrated the signal transduction role of Integrin among cells or between cells and environments, influencing the processes including cell positioning and growth, protein synthesis and energy metabolism (46). Specifically, ligands in milk (e.g., osteopontin, identified as the rabbit MFGM protein in the present study) are subject to intestinal promotion by upregulating or interacting with Integrin in gastrointestinal tracts (47, 48). This interaction might emphasize the vital role of these ligands in the functional restoration of the intestine.

According to the Huang et al. report, the AQP2 protein was present in rabbit whey (36), which was seldom reported as an MFGM protein. In this study, we reported three other AQP proteins presented in rabbit MFGM proteins for the first time. Except for AQP5, the up-regulated DEMPs in RC, AQP1, and AQP10 were also detected as the rabbit MFGM proteins in this study (Supplementary Table S2). Several reports have demonstrated the abundant expression and localization of AQP4 and AQP5 in the small and large intestine of colostrum-suckling buffalo calves (49, 50), and the reciprocity of AQP1, AQP3, and AQP5 in the mammary gland during pregnancy and lactation (51), implying the involvement of AQPs in intestinal development of offspring and milk secretion of female mammals. For the significantly up-regulated MFGM protein in rabbit mature milk, in this study, we found that the extracellular matrix proteoglycan SPOCK1 is significantly down-regulated in RC relative to RM (Table 1). Interestingly, abundant SPOCK1 was also found in the rabbit whey of RM (36), consistent with this report. At the molecular biology level, it has been fully expounded that SPOCK1 can activate PI3K/Akt, mTOR/S6K, and Wnt/β-catenin to modulate oncogenic processes comprising epithelial-to-mesenchymal transition (EMT), invasion, and migration (52); however, its function in regulating neonate development still needs to be explored. In summary, the DEMPs in rabbit milk at different lactation stages displayed the specific nutrient requirements for neonatal rabbits from colostrum or RM after delivery.

We conducted the advanced analysis (GO, KEGG, and PPI) to further reveal the functional differences of MFGM proteins in RC and RM according to the DEMPs. As for GO analysis, several terms including proteolysis, cell adhesion and ion transport were remarkably enriched in BP subgroup according to all DEMPs or up-regulated DEMPs in RC (Supplementary Tables S4, S5). Ion transport is integral to life, which involves many biochemical processes, including proteolysis and cell adhesion. Proteolysis is the process in which the protein is hydrolyzed by various proteases, resulting in the deconstruction of the substrates to peptides and/or amino acids (53). Studies have demonstrated that numerous proteolytic systems exist in milk and are strongly associated with the milk protein digestion, producing specific peptides in digestive tract (54). In consistent with these results, in the present study, 29 up-regulated DEMPs in RC compared to RM, such as Proteasome subunit beta (PSMB) and PIGK, were enriched in the biological function of proteolysis, underlying its critical impacts on neonatal rabbits after delivery. We speculate that activated proteolysis in RC may involve in the primary construction of immune system, thus protecting the gut development. For the item of Cell adhesion with 13 up-regulated DEMPs in RC, the core MFGM proteins in human (40), cattle (32) and donkey (26), have been reported to gather in this vital process. Cell adhesion is a basic biological event for the subsequent cellular behaviors such as migration, proliferation, cell cycle, and the initiation of tissue formation (55). For the gut development in rabbits, Cell adhesion supported by colostrum may enhance the intestinal barrier and function to increase neonatal viability. In line with this, as the major components of cell adhesion, vital Integrin-related proteins (Integrin subunit alpha 3, Integrin subunit alpha 2, Integrin subunit alpha V, Integrin subunit alpha 1, and Integrin alpha-2 domain-containing protein) exhibited higher levels in RC compared to RM, highlighting their important roles. Integrin signaling, triggered by binding to the extracellular matrix, orchestrates key pathways such as focal adhesion kinase (FAK) to support cell proliferation and tissue maturation (56). This mechanism might be especially critical in neonatal rabbits, whose immature organs at birth necessitate rapid postnatal development—particularly of structures like the intestinal epithelium and skin—to ensure development. Integrins are fundamental mediators of leukocyte adhesion and trans-epithelial migration, playing critical roles in early immune defense. Integrin α6 is expressed on lymphocytes and monocytes and facilitates their transmigration via binding to laminin in the basement membrane (57, 58). This is particularly relevant in neonatal immunity, as colostrum in rabbits contains abundant leukocytes and membrane fragments. These integrin-bearing cells home to the neonatal gut mucosa, thereby enhancing mucosal immune protection (58, 59). Additionally, integrin-mediated activation of FAK and Src signaling cascades coordinate mechanism of cellular trafficking and immune activation providing essential immune support to neonates with immature defense systems (60). Combining these results, we captured the functional and structural differences of MFGM proteins in RC and mature milk, which promoted an understanding of rabbit lactation.

As for the KEGG analysis, most significant pathways were enriched in ECM-receptor interaction, Cholesterol metabolism, Proteasome, PI3K-Akt signaling pathway and Human disease (Supplementary Tables S7–S9). Mounds of Human Diseases have been enriched in MFGM proteins from various mammals (human, pig, goat, and camel), followed by Metabolism and other processes (12, 24, 25, 34). In line with this, several Human Diseases were also enriched in rabbit DEMPs. Lysosome and proteasome are the two ways to eliminate the abandoned proteins, which is so-called proteolysis (53). Mounting evidence demonstrated that disorders of cell homeostasis concerning endo-lysosomal dysfunction (e.g., processing and trafficking of proteins, lipids, and other metabolites) are the key inducers for neuropathic diseases (61) and cardiomyopathy (62), Alzheimer’s disease, Parkinson’s disease, and Fabry disease, for instance. These findings enlighten the relations between rabbit milk and diseases. Moreover, abnormalities of PCD (e.g., necroptosis and ferroptosis) (63), autophagy (e.g., phagosome) (64) and signal transduction (e.g., PI3K-Akt signaling pathway) (65) might be the underlying mechanism contributing to these diseases and needs further validation.

As for the PPI analysis, seven Integrin-related proteins were acquired from rabbit milk in the present study, and their roles in cell-adhesion, signal transduction, and recognition depending on metal ion (Mg^2+^ and Ca^2+^) are well-documented (46). Although the existence of integrins as MFGM proteins in human, bovine, and donkey milk has been reported (11, 33, 41), the variety and quantity of these proteins identified from rabbit MFGM proteins in this study, especially in RC, are obviously higher than in other species, thereby characterizing the rabbit milk. As a large molecule glycoprotein in the extracellular matrix and basement membrane, FN1 is widely expressed in various animal cells, which plays a core role in cell adhesion to modulate cell growth, migration, differentiation, and polarity (66). Previous studies also found that FN1 was identified as the MFGM protein and highly expressed in goat colostrum, which might work as an acute inflammatory response protein (35). Interestingly, unlike most DEMPs that were highly expressed in RC, FN1 was highly expressed in RM, which is contrary to the previous results (35). Those results emphasized the core role of DEMPs (e.g., Proteasome subunits and Integrin) on Human diseases (e.g., Alzheimer’s disease) and Signal transduction (e.g., PI3K-Akt signaling pathway). They highlighted the valuable role of milk in rabbit development.

While this study provides novel insights into the rabbit milk and its possible impact on offspring development and health, limitations remain regarding commercial application and mechanistic evidence. Although mass differential MFGM proteins were identified from different lactating phases of rabbits for the first time, thereby improving understanding of milk components in mammals, their use in human life remains far off. Moreover, the underlying molecular mechanism of MFGM proteins on neonatal development and health is still lacking. Further investigation should focus on dissecting the interrelationships between these DEMPs and enriched pathways through targeted experimental approaches.

To summarize, for the newborn rabbits, the loss of maternal immune protection at birth necessitates the rapid establishment of their own immune defenses. Consequently, based on the results of this study, we speculated that the association of many MFGM proteins in RC with diseases likely serves a role in passive immunity, protecting infants from infections. Once neonates survive the vulnerable early postnatal period, their nutritional needs become paramount. Therefore, MFGM proteins in RM are more engaged in the nutritional metabolism, thereby better providing the basic needs for infant growth and development.

## 5 Conclusion

The rabbit MFGM protein profile was first depicted in the present study. In addition to the typical MFGM proteins, such as PLIN2 and Apolipoprotein, novel MFGM proteins were identified, enlightening the understanding of trace proteins in rabbit milk. Specifically, advanced analysis (GO/KEGG/PPI) revealed the functional differences between RC and mature milk, involving proteolysis, cell adhesion, and ion transport, and emphasizing the core role of DEMPs (e.g., Proteasome subunits and Integrin) on Human diseases (e.g., Alzheimer’s disease) and Signal transduction (e.g., PI3K-Akt signaling pathway). These results suggested the supporting roles of RC on passive immunity and RM on nutritional metabolism.

## Data Availability

The original contributions presented in the study are publicly available. This data can be found here: https://doi.org/10.17632/pv43j4jb94.1.

## Footnotes

1
https://geneontology.org/

2
https://www.genome.jp/kegg/

3
https://string-db.org
