# Supplementary material for: Data-independent acquisition quantitative proteomics analysis of milk fat globule membrane proteins in rabbit colostrum and mature milk
Source: Front Vet Sci. 2026 Mar 19;13:1703387. doi: 10.3389/fvets.2026.1703387 (PMC13045526; doi:10.3389/fvets.2026.1703387)
Supplement: Supplementary file 2 [file Data_Sheet_1.docx]

**
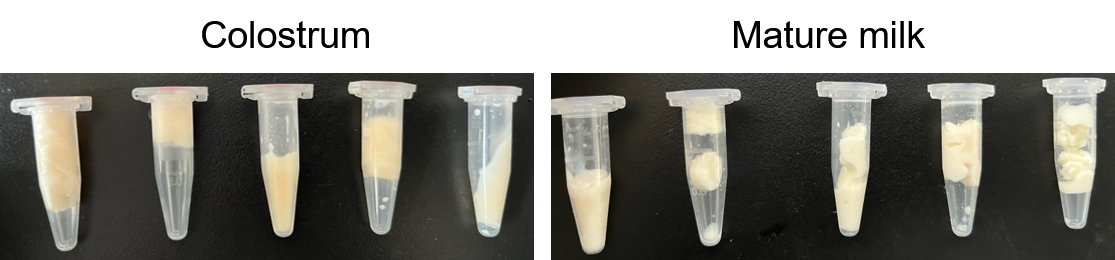
Figure S1. Representative images of frozen rabbit colostrum (RC) and mature milk (RM).**

**
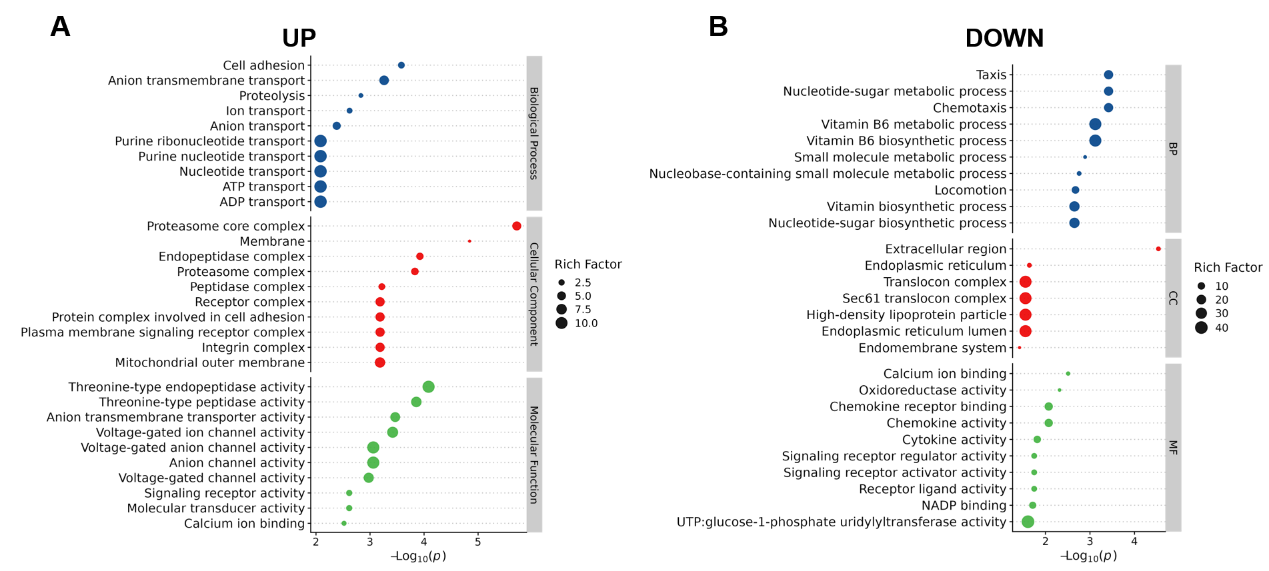
Figure S2. GO advanced analysis of up-regulated (A) and down-regulated (B) differentially expressed MFGM proteins (DEMPs) in RC and RM.**

**
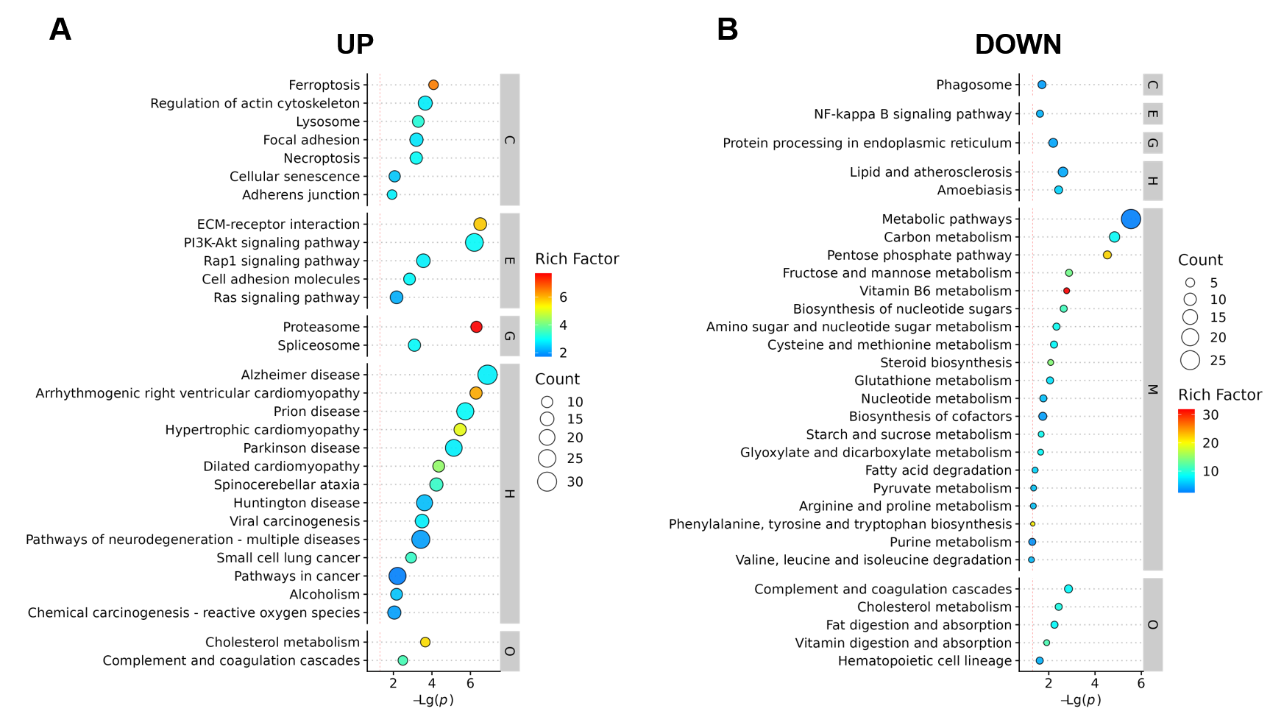
Figure S3. KEGG pathway analysis of up-regulated (A) and down-regulated (B) DEMPs in RC and RM.**

**
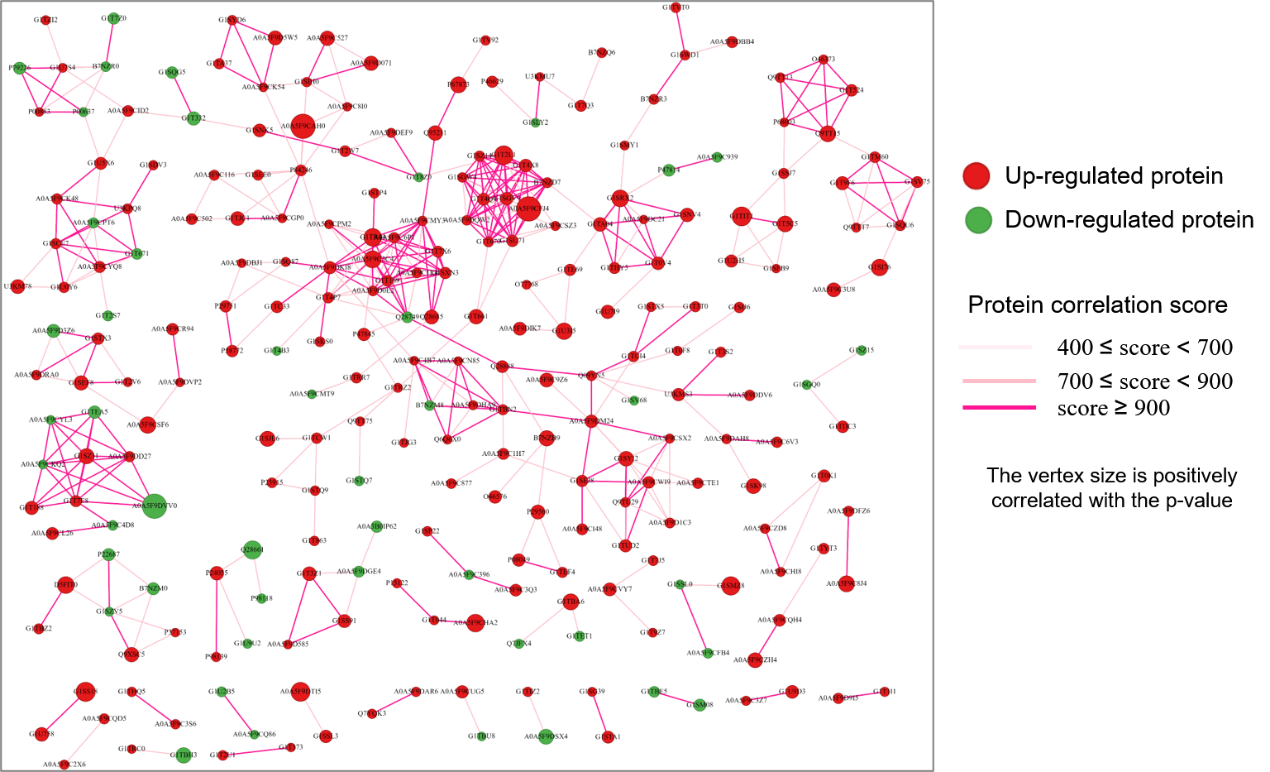
Figure S4. Protein-protein interaction (PPI) network analysis of DEMPs in RC and RM.**
